# Attention Deficit Hyperactivity Disorder (ADHD) among longer-term prison inmates is a prevalent, persistent and disabling disorder

**DOI:** 10.1186/1471-244X-10-112

**Published:** 2010-12-22

**Authors:** Ylva Ginsberg, Tatja Hirvikoski, Nils Lindefors

**Affiliations:** 1 Department of Clinical Neuroscience, Division of Psychiatry, Karolinska Institutet, Stockholm, Sweden; 2 Karolinska Institutet Center of Neurodevelopmental Disorders, Stockholm, Sweden; 3 Department of Molecular Medicine and Surgery, Center for Molecular Medicine, Karolinska Institutet, Stockholm, Sweden

## Abstract

**Background:**

ADHD is a common and disabling disorder, with an increased risk for coexisting disorders, substance abuse and delinquency. In the present study, we aimed at exploring ADHD and criminality. We estimated the prevalence of ADHD among longer-term prison inmates, described symptoms and cognitive functioning, and compared findings with ADHD among psychiatric outpatients and healthy controls.

**Methods:**

At Norrtälje Prison, we approached 315 male inmates for screening of childhood ADHD by the Wender Utah Rating Scale (WURS-25) and for present ADHD by the Adult ADHD Self-Report Screener (ASRS-Screener). The response rate was 62%. Further, we assessed 34 inmates for ADHD and coexisting disorders. Finally, we compared findings with 20 adult males with ADHD, assessed at a psychiatric outpatient clinic and 18 healthy controls.

**Results:**

The estimated prevalence of adult ADHD among longer-term inmates was 40%. Only 2 out of 30 prison inmates confirmed with ADHD had received a diagnosis of ADHD during childhood, despite most needed health services and educational support. All subjects reported lifetime substance use disorder (SUD) where amphetamine was the most common drug. Mood and anxiety disorders were present among half of subjects; autism spectrum disorder (ASD) among one fourth and psychopathy among one tenth. Personality disorders were common; almost all inmates presented conduct disorder (CD) before antisocial personality disorder (APD). Prison inmates reported more ADHD symptoms during both childhood and adulthood, compared with ADHD psychiatric outpatients. Further, analysis of executive functions after controlling for IQ showed both ADHD groups performed poorer than controls on working memory tests. Besides, on a continuous performance test, the ADHD prison group displayed poorer results compared with both other groups.

**Conclusions:**

This study suggested ADHD to be present among 40% of adult male longer-term prison inmates. Further, ADHD and coexisting disorders, such as SUD, ASD, personality disorders, mood- and anxiety disorders, severely affected prison inmates with ADHD. Besides, inmates showed poorer executive functions also when controlling for estimated IQ compared with ADHD among psychiatric outpatients and controls. Our findings imply the need for considering these severities when designing treatment programmes for prison inmates with ADHD.

## Background

ADHD is a common, inherited and disabling developmental disorder with early onset. Most often ADHD persists across the life span, affecting 2-4% of adults [1]. The core symptoms of ADHD are inattention, hyperactivity and impulsivity. Further, deficits in executive functioning are commonplace, such as planning, organising, exerting self-control, working memory, and affect regulation. Therefore, ADHD affects educational and occupational performances, psychological functioning, and social skills. Adults with ADHD are at increased risk for unemployment, sick leave, coexisting disorders, abuse, and antisocial behaviour leading to conviction [2,3]. Nearly 80% of adults with ADHD present with at least one coexisting psychiatric disorder [3,4]. Further, studies display ADHD to be common among prison inmates [5-9]. However, little attention has been paid to profiles of ADHD symptoms and executive functions of prison inmates compared with other groups affected by ADHD, and to controls [10]. Besides, effects of pharmacological treatment for ADHD among prison inmates remain unexplored. The clinical presentation has shown to change with age, as hyperactivity declines, whereas inattention and executive dysfunction persist, thus representing the core features of adult ADHD [11,12]. However, most previous studies have excluded prison inmates, questioning how relevant these findings are to prison inmates. To gain some more information, we evaluated ADHD and criminality. The first aim of this study was to estimate the prevalence of ADHD among longer-term inmates of a high-security Swedish prison. The second aim was to describe ADHD, coexisting disorders, and executive functions among prison inmates. The final aim was to compare these findings with ADHD psychiatric outpatients and healthy controls.

We hypothesized that ADHD would be common among this group comprising mainly longer-term prison inmates, typically convicted of crimes because of violence and drugs. Also, we hypothesized that they would present more severe ADHD symptoms across the lifespan, more common coexisting psychiatric disorders, and poorer executive functions compared with the other groups.

## Methods

The present study included an estimation of the prevalence of ADHD among longer-term prison inmates. Further, it included a description of ADHD and executive functions among prison inmates compared with ADHD among psychiatric outpatients and healthy controls. The Regional Ethical Board in Stockholm approved the studies. Participants provided written informed consents before study procedures.

### Participants

Norrtälje Prison is a high-security prison placed outside Stockholm, Sweden, serving the entire country, hosting 200 adult male inmates. The prison holds mainly longer-term inmates, typically convicted of crimes because of drugs or violence.

**Figure 1:**
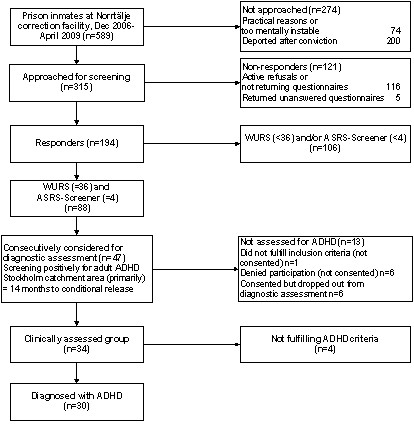
**Flow chart of the screening procedures and diagnostic assessments**.

Figure 1 shows the study flowchart. Norrtälje Prison hosted 589 inmates between December 2006 and April 2009. Of those inmates, we did not invite 200 for screening, as we could not include them in the following trial because of deportation out of the country after served conviction. Further, we did not approach 74 inmates because of practical reasons, or if we considered them as too mentally affected to take part. Thus, a specially trained correction officer successively approached 315 prison inmates for screening during the study period. Another purpose of screening was to identify subjects for a diagnostic evaluation for ADHD before recruitment for a clinical trial. Therefore, we ended recruitment as we had randomised all 30 subjects for the trial in April 2009.

Following the screening survey, we performed extensive diagnostic assessments for ADHD and coexisting disorders among a group of inmates. We selected subjects first according to their origin, as the Stockholm County Council funded the assessments as part of regular clinical practice. Thus, we invited all prison inmates marking adult ADHD by the screening, registered in the Stockholm County, with at least 14 months left to conditional release, and approved by the security officers to stay at the ADHD ward. By this pre-screening, we evaluated if subjects with ADHD would fulfil criteria for taking part in the following clinical trial with methylphenidate (Ginsberg and Lindefors, unpublished data). Subjects with coexisting disorders, such as ASD, anxiety and depression could take part if considered stable by the investigator at the assessment. Further, the general cognitive functioning had to be above the level of mental retardation. In addition, subjects could continue stable pharmacological treatment for coexisting disorders if we did not suspect treatment interfering with methylphenidate. Additionally, subjects had to be free from serious medical illnesses. Thus, after meeting criteria for the following trial and providing a written informed consent, the subject could take part in the diagnostic evaluation.

We considered 47 prison inmates for assessment. However, we excluded one subject because of an exclusion criterion, whereas six subjects denied taking part. Of 40 consented subjects, six dropped out during the assessments. Therefore, we finally assessed 34 subjects and could confirm ADHD among 30 of them (Figure 1). When appropriate, we extended the evaluation to confirm ASD in consistence with DSM-IV. We defined ASD as fulfilling the criteria for Autistic syndrome, Asperger syndrome or Pervasive developmental disorder, not otherwise specified (PDD-NOS). This evaluation included the Asperger Syndrome Screening Questionnaire (ASSQ) [13], the Diagnostic Interview for Social and Communication Disorders (DISCO) [14,15], and the Autism Diagnostic Observation Schedule (ADOS), module 4 [16].

The psychiatric outpatient study group comprised 20 adult men with ADHD, 18 of them with ADHD of the combined type, and two with the predominantly inattentive subtype. We consecutively recruited these subjects to another study [17] between 2004 and 2006, from the Neuropsychiatric Unit, Karolinska University Hospital; a psychiatric outpatient tertiary unit specialised in ADHD. Notably, the exclusion criteria for taking part were different among psychiatric outpatients, as ongoing pharmacological treatment for coexisting disorders, APD, ASD, 70 > IQ < 85, or pure 'sluggish, inattentive' ADHD [18,19] excluded. Because of different criteria, we expected a difference in IQ between groups. Thus, we controlled for IQ in the statistical analyses of executive functions.

The control group [17] comprised 18 adult healthy males not needing psychiatric care, assessment for learning difficulties or educational support during childhood. Further, they did not need psychiatric care during the present study. We recruited age-matched controls from advertisement on fitness training centres in Stockholm City and among friends of staff-members.

### Procedures

#### Estimation of ADHD prevalence among longer-term prison inmates

WURS is a 61-item self-administered scale for rating frequencies of ADHD childhood symptoms and behaviours retrospectively on a 5-point scale, from 0 = *not at all or slightly*, to 4 = *very much*. The subscale WURS-25 provides a total sum score (range 0-100) by summing those 25 items best discriminating between ADHD and controls [20]. According to the originators, a cut-off score of 36 is 96% sensitive and specific for identifying childhood ADHD among the general population [20].

The ASRS-Screener comprises the 6 out of 18 most predictive items of the Adult ADHD Self-Report Scale (ASRS) [21] for defining present ADHD in adulthood. Fulfilling at least 4 out of 6 significant items [22] on ASRS-Screener defines adult ADHD. Both scales are standard tools in clinical practice, despite the lack of Swedish validations. In this study, we defined adult ADHD as reaching the cut-off levels for WURS-25 and ASRS-Screener, respectively.

#### Assessment for ADHD among prison inmates

Board certified psychiatrists and clinical psychologists well experienced in ADHD, conducted the clinical assessments. We confirmed ADHD in accordance to DSM-IV [23]. The evaluations included a semi-structured clinical diagnostic interview for ADHD based on the DSM-IV-criteria [23]. Further, ASRS [24] is an 18-item self-administered scale with appropriate psychometric properties [25] based on the DSM-IV criteria and adjusted to reflect ADHD symptoms as seen in adults [22]. We used a non-validated Swedish version of the ASRS [24] for rating symptom frequencies on a 5-point scale, from 0 = *never*; to 4 = *very often*, providing a total sum score (range 0-72).

Whenever possible, we collected collateral information from parents or other significant others by questionnaires, before psychologists or psychiatrists performed interviews. The questionnaires included the Five to Fifteen (FTF) questionnaire [26,27] and the Conners' Brief Parent Rating Scale - Conners' Hyperactivity Index [28,29], respectively.

*The Five to Fifteen (FTF) questionnaire *[26,27] elicits childhood symptoms and developmental problems of ADHD and coexisting disorders in the ages five to fifteen years. The FTF shows acceptable to excellent inter-rater and test-retest reliability and comprises 181 items scored on a 3-point scale, from 0 = *does not apply*, to 2 = *definitely applies*.

*The Conners' Brief Parent Rating Scale - Conners' Hyperactivity Index *is validated in several countries. This scale describes ADHD and oppositional defiant symptoms and behaviours in children up to 10 years of age [28], comprises 10 items, scored 0-3, and provides a total sum score (0-30).

We collected additional collateral information by medical records from child- and adolescent psychiatry, school health services, adult psychiatry and forensic psychiatry. Further, we evaluated coexisting disorders by the Structured Clinical Interview for DSM-IV Axis I Disorders (SCID I) [30], the Hare Psychopathy Check List-Revised (PCL-R), a semi-structured interview defining psychopathy by a total sum-score ≥ 30 [31], and the self-rated version of the Structured Clinical Interview for DSM-IV Axis II personality disorders, the SCID II Patient Questionnaire (SCID II PQ). We estimated frequencies of personality disorders by increasing the screening cut-off level for each personality disorder by one score. This procedure has shown an acceptable agreement with the SCID II interview [32]. Furthermore, the evaluation comprised a medical history, physical examination, routine laboratory tests, urine drug screening and a neuropsychological test battery assessing IQ and executive functions. As prison inmates often present learning disabilities such as reading difficulties [9], we assessed neuropsychological tests not requiring reading, writing or mathematic skills. We estimated IQ by the *Wechsler Adult Intelligence Scale-III *subtests Vocabulary and Block Design, a dyadic short form correlating 0.92 with WAIS-III FSIQ [33,34].

#### Neuropsychological tests of executive functions

Digit Span [33] measures verbal working memory (WM) whereas Span Board [35] measures visuospatial WM. Further, we measured sustained attention, impulse inhibition and other executive functions by the computerized *The Conners' Continuous Performance Test II (CCPT) *[36]. The CCPT measure Hit RT reflects *basic reaction time*, whereas Hit RT SE, Variability, Hit RT block change, Hit SE block change, Hit RT ISI change, Hit SE ISI change and Perseverations reflect *variability dependent measures*. Finally, Omission errors, Commission errors, Detectability (d'), and Response style (â) reflect *accuracy dependent measures*.

#### Assessment for ADHD among psychiatric outpatients

The diagnostic evaluation comprising neuropsychological tests was similar as among prison inmates. However, we did not assess SCID I, SCID II PQ, or PCL-R among ADHD psychiatric outpatients. Case files provided information on psychiatric comorbidity. Besides, the self-rated Beck Depression Inventory [37,38], the Beck Anxiety Inventory [39], and the Current ADHD Symptom Scale - Self-Report Form [40], evaluated present psychiatric symptoms.

#### Healthy controls

We interviewed controls for confirming the absence of learning difficulties or psychiatric problems during childhood and the study, respectively. Further, we used the same self-rating scales for present psychiatric symptoms as among the psychiatric outpatients. Finally, the neuropsychological tests were similar as for the other groups.

### Statistical analysis

Descriptive statistics summarised demographic data and clinical characteristics of subjects. We carried out inferential statistics by analyses of variance (ANOVA), Student's t-test or Mann-Whitney *U*-test for continuous measures, and chi-square test or Fisher's exact test for categorical measures. Further, for comparing between groups on neuropsychological measures, we performed a series of analysis of variance (ANOVA) with Bonferroni corrected post hoc comparisons, whenever main analyses reached significance. In addition, we aimed to control for IQ differences. Thus, we reanalysed measures of executive functions (DS, SB, and CCPT) by performing a series of ANCOVA with the dyadic estimated IQ entered as a covariate. By these analyses, we evaluated if lower IQ among prison inmates could explain their executive dysfunctions. We present statistics from both ANOVAs and ANCOVAs, as most measures of executive functions did not co-vary with IQ. We set the alpha-level at *p *= .05. Finally, we performed all statistical analyses by SPSS 17.0 and 18.0, respectively.

## Results

### ADHD prevalence

Figure 1 presents a flowchart of the study. As calculated from this figure, the total response rate was 62% (194/315). We defined adult ADHD as reaching the cut-off levels for both childhood and adult ADHD. By this procedure, we increased the specificity of the screening survey. When applying our predefinition of adult ADHD, the prevalence rate was 45%, as 88 out of 194 subjects fulfilled this definition (Figure 1). Overall, responders were slightly older and served longer convictions compared with non-responders (Table 1). However, when we assessed 34 subjects marking ADHD by the screening, we confirmed ADHD among 30 of them. Thus, the screening survey pointed out to be 88% (30/34) specific. Therefore, we imply a more conservative 40% ADHD prevalence (0.88 × 45) among longer-term prison inmates.

**Table 1:** Demographic and Clinical Characteristics of Prison Survey Sample

*Study sample (n = 315)*	*Responders* *(n = 194)*	*Non* *responders^a^* *(n = 121)*	*p*
**Men, n (%)**	194 (100)	121 (100)	

**Age, median^b ^(IQR), y**	31.3 (14)	29.4 (12)	.028^d^

**Conviction time, median^b ^(IQR)^c^, months**	69 (66)	60 (54)	.030^d^

^a^Non-responders were defined as those approached but actively refused to take part, those who consented but not returned questionnaires, and those who returned unanswered questionnaires; ^b^Medians were used as measures of central tendencies as age and conviction time were non-normally distributed; ^c ^IQR: Interquartile range; ^d^Mann-Whitney *U*-test was employed due to non-normal distributed data.

### Clinical characteristics of ADHD among adult male prison inmates

This study included an extensive diagnostic evaluation of ADHD and coexisting disorders among a group of prison inmates (Figure 1). Table 2 shows the clinical characteristics of those 30 subjects confirmed with ADHD. As shown, almost all subjects confirmed ADHD of the combined type. Further, all subjects presented coexisting disorders. In fact, all 30 subjects presented a lifetime history of SUD, with amphetamine as the most preferred drug among almost two thirds. In general, the subjects showed an early onset of abuse and antisocial behaviour. In addition, lifetime mood and anxiety disorders were obvious among a vast majority and treated among almost half of subjects at the assessment. Besides, almost one fourth confirmed ASD, much more common than we expected. On the other hand, psychopathy was present among only one tenth, which was less than we expected. Further, personality disorders were present among 96% (22/23) of subjects. Among personality disorders, antisocial, borderline, paranoid, narcissistic, or obsessive-compulsive personality disorder were most obvious. Further, there was a striking finding of this study; despite most subjects reported prior need of health services and educational support at school, few received a diagnosis of ADHD during childhood. In summary, prison inmates showed severe symptoms and severities from ADHD, SUD, ASD, personality disorders, mood- and anxiety disorders.

**Table 2:** Demographic and Clinical Characteristics of Assessed Groups; ADHD-prison group, ADHD-psychiatry group, Healthy controls. Not applicable = N/A.

	*ADHD-prison, n = 30*	*ADHD-psychiatry, n = 20*	*Controls*, *n = 18*	*F or* *χ^2^*	*p*
**Age, mean, (SD), y**	34.4 (10.67)	33.4 (8.65)	35.2 (9.85)	.14	.87^e^

**Educational level, nine-year compulsory school or less, n (%)**	25 (83)	6 (30)	1 (6)	39.28	< .001^e^

**ADHD, combined, n (%)**	28 (93)	18 (90)	N/A		

**ADHD, inattentive, n (%)**	2 (7)	2 (10)	N/A		

**≥ 1 current co-morbid disorder, n (%)^a^**	15 (50)	12 (60)	N/A		.569

**Autism spectrum disorder^b^**	7 (23)	N/A	N/A		

**Mood and anxiety disorder, lifetime^a^**	22 (73)	N/A	N/A		

**Personality disorders, (N = 23)^c^**					

**Antisocial, n (%)**	22 (96)	N/A	N/A		

**Borderline, n (%)**	17 (74)	N/A	N/A		

**Paranoid, n (%)**	17 (74)	N/A	N/A		

**Narcissistic, n (%)**	15 (65)	N/A	N/A		

**Obsessive-Compulsive, n (%)**	12 (52)	N/A	N/A		

**Passive-Aggressive, n (%)**	11 (48)	N/A	N/A		

**Avoidant, n (%)**	11 (48)	N/A	N/A		

**Depressive, n (%)**	8 (35)	N/A	N/A		

**Dependent, n (%)**	7 (30)	N/A	N/A		

**Schizotypal, n (%)**	5 (22)	N/A	N/A		

**Schizoid, n (%)**	2 (9)	N/A	N/A		

**Histrionic, n (%)**	0 (0)	N/A	N/A		

**Substance use disorder, n (%)^a^**	30 (100)	N/A	N/A		

**Amphetamine preferred, n (%)**	19 (63)	N/A	N/A		

**Cocaine preferred, n (%)**	4 (13)	N/A	N/A		

**Alcohol preferred, n (%)**	4 (13)	N/A	N/A		

**Psychopathy, n (%)^d^**	3 (10)	N/A	N/A		

**Concomitant psychotropic's, n (%)**	13 (43)	N/A	N/A		

**Onset of alcohol, mean (SD), y**	11.9 (1.81)	N/A	N/A		

**Onset of illegal drugs, mean (SD), y**	14.0 (2.41)	N/A	N/A		

**Onset of criminality, mean (SD), y**	11.2 (3.40)	N/A	N/A		

**Educational assistance at school, n (%)**	24 (80)	N/A	N/A		

**Child psychiatry/school health, n (%)**	18 (60)	N/A	N/A		

**ADHD diagnosed in childhood, n (%)**	2 (7)	N/A	N/A		

^a^According to DSM-IV by the SCID I interview, ^b^According to DSM IV, Autism spectrum disorder includes both Asperger syndrome and PDD-NOS, ^c^Frequencies of personality disorders were estimated by increasing the cut-off level for each personality disorder by one score, on the SCID II PQ to equal the cut-off score of the SCID II interview, ^d^Psychopathy was defined as a total sum score of ≥30 by the PCL-R, ^e^Analyses of variance (ANOVA) for continuous variables and Fisher's exact test for categorical variables.

### Comparisons between ADHD prison inmates, ADHD psychiatric outpatients, and healthy controls

As depicted in Table 2, all three groups were of similar age. Notably, 83% of ADHD prison inmates fulfilled nine-year of compulsory school or less, compared with 30% among ADHD psychiatric outpatients, and 6% among healthy controls, thus reflecting a remarkably lower educational level among prison inmates.

### Standardised questionnaires

**Figure 2:**
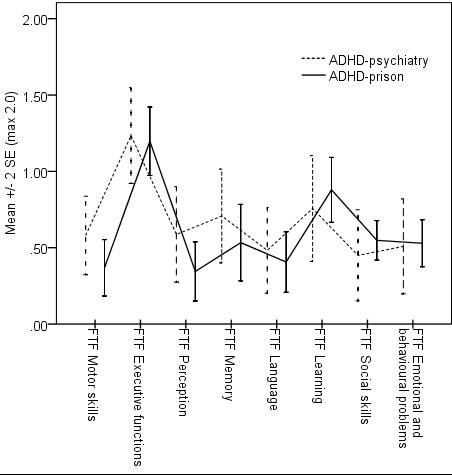
**Retrospective ratings of childhood symptoms by the Five to Fifteen questionnaire as completed by significant others, for the ADHD-psychiatry group (n = 15) and the ADHD-prison group (n = 14), respectively**.

The ADHD-prison group rated more ADHD related symptoms and behaviours during both childhood and adulthood, compared with the ADHD-psychiatry group (Table 3). By contrast, when parents retrospectively rated childhood symptoms and behaviours, differences between groups were negligible, which we did not expect. Table 3 presents statistics and Figure 2 presents mean values (+/- 2 SE), respectively.

**Table 3:** Self-rated ADHD symptoms and behaviours during both childhood and adulthood; parental ratings of childhood ADHD-symptoms. All results divided by group.

	*ADHD-psychiatry* *n = 20*	*ADHD-prison n = 30*	*t*	*p*
***Self-rating questionnaires***	*M (SD)*	*M (SD)*		

**WURS-25**	54.70 (14.31)	67.43 (13.48)	-3.19	.002

**ASRS^a^**	45.11 (12.85)	55.30 (8.89)	-3.28	.002

***Parental rating/questionnaires completed by significant others***				

**Five to Fifteen - Executive Functions Subscale^b^**	1.23 (0.59)	1.20 (0.44)	0.19	.848

**Conners' Hyperactivity Index^b^**	13.47 (10.34)	15.19 (8.07)	-0.52	.608

^a ^Data missing for one subject among the ADHD-psychiatry group; ^b ^The FTF Executive Functions Subscale includes ADHD criteria according to DSM-IV. For 15/20 (75%) among the ADHD-psychiatry group and 16/30 (53%) among the ADHD-prison group, a significant other completed the FTF and the Conners' Hyperactivity Index. For all questionnaires, higher scores indicate increased problems.

### Neuropsychological tests

The dyadic estimation of IQ displayed similar IQ for controls and the ADHD-psychiatry group; (Controls, n = 18, M = 112 (± 9.65), range 97 - 132); (ADHD-psychiatry, n = 20, M = 108.25 (±11.48), range 89 - 132). On the other hand, IQ was substantially lower among ADHD prison inmates; (M = 95.18 (± 9.99), range 78 - 113). The ADHD-prison group (n = 22) had missing data for eight subjects. We expected significant differences between groups on estimated IQ (*F *= 14.76, *p *< .001, *η*_p_^2 ^= .341) because of different inclusion criteria. In fact, only the ADHD-prison group included subjects with IQ between 70 and 85. As a result, 10% (3/30) of prison inmates presented estimated dyadic IQ within this range, specifically between 78 and 85. Therefore, we excluded those three inmates with IQ < 85 for making inclusion criteria homogenous. However, the ADHD-prison group still showed lower estimated IQ after performing this procedure, compared with both other groups (*F *= 10.49, *p *< .001, *η*_p_^2 ^= .28).

#### Neuropsychological tests of executive functions

The ADHD-prison group showed poorer results on several measures of executive functions compared with both other groups, also when controlling for IQ (Table 4).

**Table 4:** ANOVA statistics included post hoc IQ adjustments for tests of executive functions. The statistics F, p, and ηp2 presented for ANOVAs without IQ adjustments. On working memory tests, higher scores reflect better results, whereas on Conners' CPT II, higher scores reflect poorer results.

*Test and measured function*	*N*	*F*	*p*	***η***_**p**_^**2**^	*Post hoc test*	*Post hoc adjusted for IQ*
***Measures of working memory***	Control:18 ADHD-psych: 20 ADHD-prison: 30					

***Digit Span***		21.29	< .001	.396	C>Psych > Prison	C > Psych = Prison

***Span Board***		24.88	< .001	.434	C>Psych > Prison	C > Psych = Prison

***Conners' CPT II***	Control:18 ADHD-psych: 20 ADHD-prison: 27					

**CCPT reaction time**						

**Hit RT**		.48	.617	.015	C = Psych = Prison	C = Psych = Prison

***CCPT variability***						

**Variability**		26.38	< .001	.460	C = Psych < Prison	C = Psych < Prison

**Hit RT block change**		.29	.749	.009	C = Psych = Prison	C = Psych = Prison

**Hit SE block change**		.165	.848	.005	C = Psych = Prison	C = Psych = Prison

**Hit RT ISI change**		1.22	.302	.038	C = Psych = Prison	C = Psych = Prison

**Hit SE ISI change**		.662	.519	.021	C = Psych = Prison	C = Psych = Prison

**Perseverations**		8.66	< .001	.218	C = Psych < Prison	C = Psych < Prison

***CCPT accuracy***						

**Omission errors**		16.23	< .001	.344	C = Psych < Prison	C = Psych < Prison

**Commission errors**		12.61	< .001	.289	C = Psych < Prison	C = Psych < Prison

**Detectability (d')**		9.21	< .001	.229	C < Prison Psych = C Psych = Prison	C < Prison Psych = C Psych = Prison

**Response style (beta)**		4.27	.018	.121	Psych < Prison Psych = C Prison = C	Psych < Prison Psych = C Prison = C

Note: CCPT = Conners' Continuous Performance Test; RT = reaction time; SE = standard error; ISI = interstimulus interval; N/A = not applicable

On measures of working memory, controls outperformed the ADHD-psychiatry group on both verbal (DS) and visuo-spatial working memory (SB). On the other hand, the ADHD-psychiatry group outperformed the ADHD-prison group on the same measures. However, when controlling for IQ, the differences in working memory between ADHD groups no longer remained, but controls still outperformed both ADHD groups. Thus, both working memory tests showed executive dysfunctions associated with ADHD, also when controlling for IQ.

**Figure 3:**
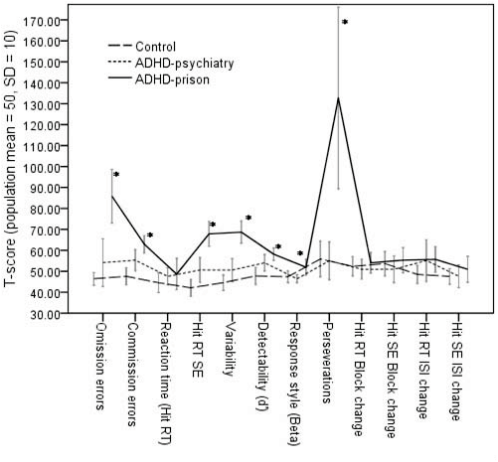
**The Conners' Continuous Performance Test II (CCPT)**. Results are presented for controls (n = 18), the ADHD-psychiatry group (n = 20), and the ADHD-prison group (n = 27), respectively. The CCPT results did not co-vary with IQ. Note: * the ADHD-prison group performed significantly poorer than at least one of the other groups (ADHD-psychiatry and controls).

On the Conners' Continuous Performance Test II (CCPT), controls and the ADHD-psychiatry group showed similar results. However, at least one of the other groups outperformed the ADHD-prison group on all four *accuracy dependent measures*, and in three out of seven *variability dependent measures*, respectively. On the other hand, there were no significant differences in *reaction time *between groups (Table 4 and Figure 3). Notably, 5 out of 27 (18.5%) subjects among the ADHD-prison group showed remarkably increased values (T-score >200) on Perseverations, a measure considered to reflect flexibility. Therefore, we performed analyses both including and excluding subjects with extreme values. However, we observed similar results on Perseverations also when excluding those subjects, thus implying decreased flexibility among prison inmates with ADHD. Further, estimated IQ did not explain the CCPT results in this study (Table 4).

## Discussion

The present study included an estimation of ADHD prevalence among adult male longer-term prison inmates from a high-security Swedish prison. Further, we evaluated ADHD and executive functions among prison inmates and then compared results with ADHD psychiatric outpatients and healthy controls. We estimated a prevalence rate as high as 40% among these prison inmates. Further, those inmates we later confirmed with ADHD were severely affected and disabled from ADHD and coexisting disorders, such as SUD, ASD, personality disorders, mood- and anxiety disorders. Previous studies reported increased frequencies of major mental disorders, personality disorders, and early adjustment problems among prison inmates, regardless of ADHD [41]. The present study confirms these observations. In addition, educational level and executive functions were poorer among ADHD inmates compared with ADHD psychiatric outpatients and controls. These findings remained after controlling for IQ. Thus, our findings imply prison inmates with ADHD to present a severely affected group of ADHD.

Although ADHD is common among prison inmates, prevalence rates are inconsistent, probably because of different used criteria among different prison populations [5-9]. Further, symptoms of ADHD, such as hyperactivity and impulsivity have shown to decline by age, whereas inattention and executive dysfunction continue [12]. Besides, most prevalence studies on male prison inmates have been conducted among younger inmates [8]. Further, knowledge is sparse on clinical features and executive functions among adult male prison inmates confirmed with ADHD [6-10] compared with adult ADHD among other groups and controls.

To our best knowledge, this study is the first to report a screening survey for ADHD, followed by extensive evaluations of ADHD and coexisting disorders among adult male longer-term prison inmates. The evaluations incorporated both self-reports and confirming collateral information from parents, medical records and school reports. Additionally, evaluations included a physical examination and neuropsychological assessments. Further, we compared ADHD prison inmates with ADHD psychiatric outpatients and controls for ADHD symptom load, coexisting disorders and executive functions.

### Prevalence of ADHD among prison inmates

As hypothesized, ADHD was prevalent among these adult male longer-term prison inmates with a median age of 31 years. We estimated the prevalence as high as 40%, compared with previous findings by Rösler et al [8] who reported a prevalence of 45%, though among younger inmates (mean age 19). Thus, our results suggest ADHD to be comparably present among older and younger inmates. Our finding contradicts the common view of ADHD to decline by age. Thus, this symptom reduction by age might not held true for ADHD prison inmates. Further, the total survey response rate was 62%, which we view as acceptable, considering a common mistrust against authorities among prison inmates. However, we have to consider the attrition rate and its impact on the results. We imply that we not exaggerated the ADHD prevalence, as we did not approach inmates who we considered too psychiatric affected to take part. In some of these cases, ADHD might contribute to their psychiatric symptoms. On the other hand, we can not exclude some selection bias at the end of the study period when the study was more commonly known in the Swedish prison and probation service. It might be that some inmates recognised themselves as having ADHD and therefore applied for serving conviction at Norrtälje Prison in hope for treatment. However, as we screened the majority at the beginning of the study period, we imply this potential bias to be of minor importance. In summary, when considering the specificity of the screening procedure, we suggest a 40% ADHD prevalence rate among adult male longer-term inmates from a high-security prison.

### Clinical characteristics of ADHD

This study only partially supported our hypothesis that ADHD prison inmates would present more severe ADHD symptoms across the lifespan, compared with ADHD psychiatric outpatients. The ADHD-prison group reported more ADHD symptoms and behaviours during both childhood and adulthood. However, collateral information from parents on childhood symptoms did not reveal any differences between groups. As a result, subjects rated more childhood symptoms retrospectively compared with parental ratings. This observation contradicts previous findings by Barkley [42] who displayed adults with ADHD to underreport their symptoms compared with parents. Thus, when considering the negative trajectory of these prison inmates and continuing ADHD symptoms, you would predict symptoms to be obvious during childhood, consistent with self-reports. Further, most subjects reported previous need of health services and educational support during childhood, pointing to obvious difficulties, although not recognised as ADHD. Notably, prison inmates showed a remarkably lower educational level compared with both other groups. Lower IQ levels among these inmates might partially explain these findings. Further, executive dysfunctions may contribute to lower school attendances and performances. In fact, we expect educational underachievement among ADHD also with normal IQ [43]. Besides, more hindering symptoms from ADHD and coexisting learning disabilities, including dyslexia and externalising symptoms such as ODD and CD, possibly contribute to poorer educational achievements and early dropouts from school. Another explanation might be prison inmates exaggerating their symptoms in hope for methylphenidate treatment. However, parents of both ADHD groups rated similarly on Conners' Hyperactivity Index. This index reflects externalising symptoms besides ADHD, which is notable considering the negative trajectory of our ADHD-prison group. Therefore, self-reported childhood symptoms by prison inmates seem more in line with their negative trajectories across time. Further, symptoms of substance abuse, depression and anxiety could mimic ADHD. However, our inmates were kept from drugs for more than three months, in some cases for years. Further, all coexisting disorders were stable and treated at the assessment, thus implying present symptoms to be ADHD related.

To summarise, our findings imply the importance of recognising ADHD early and offering effective treatment immediately. Prospective studies should evaluate if treatment will reduce the risk for serious outcomes.

### Coexisting disorders

As hypothesized, coexisting disorders were common among our prison inmates. In fact, all subjects reported a lifetime history of SUD, with amphetamine as the most preferred drug of choice. Besides, abuse and antisocial behaviour had an early onset, consistent with previous findings [44]. Additionally, anxiety disorders and depression were common, and half of inmates received treatment at the assessment. Further, all but one subject displayed CD before APD. Notably, psychopathy was present among only one tenth, which was fewer than we expected, as all but one subject displayed APD. However, previous studies reported that most psychopaths fulfil the criteria for APD, whereas the opposite is true for only a minority of inmates. These findings signal that psychopathy would be a more homogeneous disorder than APD [31]. In addition, Soderstrom used a 3-factor model of PCL-R among forensic subjects for distinguishing psychopathy traits and evaluating if certain traits reflected ADHD [45]. By this model, he showed that total PCL-R scores, as well as Factor 2 (unemotionality) and Factor 3 (behavioural dyscontrol), reflected ADHD. However, Factor 1 defining exaggerated self-opinion towards others and dishonesty did not reflect ADHD. In fact, the literature considers these interpersonal traits of Factor 1 to be most specific of psychopathy. Besides, we confirmed ASD among almost one fourth of ADHD prison inmates, mainly PDD-NOS. We are not aware of any previous reports estimating the prevalence of ASD among prison inmates. However, Anckarsater [46] showed that ASD was more common among forensic subjects than among the general population. In that study [46], PDD-NOS presented the most common ASD, paralleling our findings. In summary, we suggest that ASD is common also among prison inmates. However, studies comprising larger samples need to confirm these preliminary findings. If ASD is common among prison inmates, we need to consider this for successfully meeting the specific needs of these inmates.

Previous studies reported that personality disorders are common among different ADHD populations, such as prison inmates [9]. Recently, Rydén et al observed that personality disorders were common among adults with "pure" ADHD, ADHD combined with bipolar disorder, and bipolar disorder only, although most prevalent among "pure" ADHD (Rydén E, and collaborators, personal communication). For defining personality disorders, they used the same procedure as in the present study. By comparing those, "pure" ADHD with our ADHD prison inmates, most personality disorders implied more common among inmates. However, histrionic, depressive, and schizoid personality disorder implied more common among "pure" ADHD subjects (Rydén E, and collaborators, personal communication).

### Cognitive abilities

The present study supported our hypothesis that ADHD prison inmates would present poorer cognitive abilities compared with ADHD psychiatric outpatients and healthy controls. As expected, the ADHD-prison group showed lower estimated IQ. However, different inclusion criteria could not explain the observed IQ differences between groups, as differences remained when excluding prison inmates with IQ < 85. As presented, both ADHD groups displayed poorer executive functions compared with controls, also when adjusting for IQ. Working memory functions were similar between ADHD groups when adjusting for IQ. Considering the CCPT results overall, controls and the ADHD-psychiatry group showed similar results. Further, at least one of them outperformed ADHD prison inmates on all *accuracy dependent measures*, and on several *variability dependent measures*, respectively. On the other hand, *reaction time *was comparable between groups, thus implying slow reaction time not to be a concern among adult ADHD. Summarising, these findings are in line with theories of ADHD as an executive disorder [47]. In addition, these findings parallel recent reports by Wood et al [48,49] who suggested that lower IQ does not account for the key cognitive problems noted among ADHD. Further, one striking notion of the present study, was the increased levels of Perseverations on the CCPT, which reflects difficulties in holding back or adjusting non-proper behaviours. Previous studies reported response perseveration among ADHD subjects suffering from CD [50-52], as well as among pathological gamblers [53]. However, researchers interpreted response perseveration among ADHD in different ways. Quay [50,51] suggested increased thriving for rewards among CD, because of a more actively working behavioural activation (reward) system (BAS) compared with the behavioural inhibition system (BIS). Reverse, he suggested less active BIS compared with BAS among ADHD. Beauchaine interpreted the opposite way [54], as he suggested less active BAS among CD, resulting in a reward-seeking behaviour as a stimulation seeking. Finally, Seguin [55], Newman and Wallace [56,57] respectively, suggested deficits in attending for peripheral information, which usually directs the subject changing for a more effective behaviour. Therefore, future studies should explore the cognitive underpinnings of response perseveration, as they remain elusive.

### Limitations

We have to consider several limitations of this study. As the attrition rate of the screening survey was 38%, we must interpret the results with caution. However, we imply that we not exaggerated the prevalence rate of ADHD. Further, both rating scales used for screening lack Swedish validations. Nevertheless, these scales are used as standard tools in clinical practice. Besides, this study included only male longer-term prison inmates, why results can not extend to female inmates or to inmates serving shorter-term convicts. Further, there might have been selection bias when recruiting for the screening survey, mainly at the end of the study period when the study was commonly known in the Swedish Prison and Probation service. It might be that some inmates recognised themselves as suffering from ADHD and therefore applied for serving conviction at Norrtälje Prison in hope for treatment. However, as we screened the majority at the beginning of the study period, we imply this potential bias as minor important. Further, there might have been selection bias because of different inclusion criteria between groups. Therefore, it implies a selection of subjects among ADHD psychiatric outpatients, functioning better than average, as treatment for coexisting disorders excluded for the present study. Actually, in clinical practice, adults with ADHD often receive treatment for common coexisting disorders. On the other hand, the ADHD-psychiatry group may better reflect ADHD among the general population, as considered presenting less severe symptoms and severities compared with psychiatric outpatients. Additionally, there may have been a selection bias when recruiting prison inmates for diagnostic assessments. We noticed a few prison inmates denied taking part in the study in lack of motivation for changing their behaviour, or resistance to stay at the ADHD ward. The ward was apart from other wards for reducing the risk of exposing inmates to illicit drugs. As a result, study subjects received less time for physical exercise and restricted access to some prison programmes, as long as they stayed at the ADHD ward. Therefore, a selection bias towards more motivated prison inmates could have been present. If so, the bias probably worked towards better performances and higher functioning, than the reverse. Finally, the study samples were small. However, results were statistically significant despite small sample sizes. Notably, the strength of this study was the extensive clinical description of ADHD, coexisting disorders and executive functioning among prison inmates, as well as comparisons with ADHD psychiatric outpatients and controls. The extensive diagnostic evaluation included self-reported information, collection of collateral information, physical examination, structured diagnostic interviews and neuropsychological assessments. To our best knowledge, such extensive evaluations of longer-term prison inmates have not previously been reported. We infer our reported findings of ADHD symptom severity, coexisting disorders and executive functioning among prison inmates, are clinical important and relevant. We need to consider these severities when adjusting existing, or designing new ADHD treatment programmes for prison inmates. Further, these extensive evaluations might provide helpful insight for addressing future research on ADHD endophenotypes. Knowledge on endophenotypes may promote individually tailored treatments by identifying who will benefit from treatment. Finally, we will report effects of methylphenidate treatment among these ADHD prison inmates in another paper (Ginsberg and Lindefors, unpublished data).

## Conclusions

This study suggested ADHD to be present among 40% of adult male longer-term prison inmates. Diagnostic evaluations for ADHD among 30 inmates showed them severely disabled from ADHD and coexisting disorders, such as SUD, ASD, personality disorders, mood- and anxiety disorders. Further, these ADHD prison inmates displayed poorer executive functions, also when controlling for estimated IQ, compared with ADHD psychiatric outpatients and healthy controls. We infer the reported findings of ADHD symptom severity, coexisting disorders and executive functioning among prison inmates, are clinical important and relevant. These findings imply the need for considering these severities when introducing ADHD treatment programmes for prison inmates.

## Competing interests

YG has been on the speaker's bureau and consultant for Janssen-Cilag, Novartis and Lundbeck A/S. YG has been the principal investigator of two clinical trials sponsored by Janssen-Cilag. NL has been the investigator of a clinical trial sponsored by Janssen-Cilag. TH declares no conflicts of interest.

## Authors' contributions

YG designed the study in collaboration with NL and TH, applied to the Ethical Board in collaboration with NL, prepared the Case Report Forms, conducted clinical assessments, collected data, planned and executed the analyses, interpreted the results in collaboration with TH and NL, and prepared all drafts of the manuscript in collaboration with TH. NL revised the manuscripts critically. TH was responsible for assessments and analyses of the psychiatric outpatients and controls. All authors read, commented on and approved the final manuscript.

## Authors' information

^1 ^Department of Clinical Neuroscience, Division of Psychiatry, Karolinska Institutet, Stockholm, Sweden, ^2 ^Karolinska Institutet Center of Neurodevelopmental Disorders, Stockholm, Sweden, ^3 ^Department of Molecular Medicine and Surgery, Center for Molecular Medicine, Karolinska Institutet, Stockholm, Sweden^.^

## Pre-publication history

The pre-publication history for this paper can be accessed here:

http://www.biomedcentral.com/1471-244X/10/112/prepub
